# Transglutaminase 2 in diabetes mellitus: Unraveling its multifaceted role and therapeutic implications for vascular complications

**DOI:** 10.7150/thno.95742

**Published:** 2024-03-25

**Authors:** Kwon-Soo Ha

**Affiliations:** Department of Molecular and Cellular Biochemistry, Kangwon National University School of Medicine, Chuncheon, Kangwon-do 24341, Korea.

**Keywords:** diabetic complications, hyperglycemic memory, inhibitors, transglutaminase 2, vascular leakage

## Abstract

Diabetes, a severe metabolic disease characterized by chronic hypoglycemia, poses debilitating and life-threatening risks of microvascular and macrovascular complications, including blindness, kidney failure, heart attacks, and limb amputation. Addressing these complications is paramount, urging the development of interventions targeting diabetes-associated vascular dysfunctions. To effectively combat diabetes, a comprehensive understanding of the pathological mechanisms underlying complications and identification of precise therapeutic targets are imperative. Transglutaminase 2 (TGase2) is a multifunctional enzyme implicated in the pathogenesis of diverse diseases such as neurodegenerative disorders, fibrosis, and inflammatory conditions. TGase2 has recently emerged as a key player in both the pathogenesis and therapeutic intervention of diabetic complications. This review highlights TGase2 as a therapeutic target for diabetic complications and explores TGase2 inhibition as a promising therapeutic approach in their treatment.

## 1. Introduction

Diabetes, a serious metabolic disorder arising from chronic hypoglycemia, induces progressive vascular damage and dysfunction, leading to both microvascular and macrovascular complications 1, 2. Microvascular complications include diabetic retinopathy (DR), diabetic nephropathy (DN), diabetic peripheral neuropathy, and diabetic pulmonopathy (DP), arising from chronic hyperglycemia affecting the retina, renal glomerulus, peripheral nerves, and lungs, respectively 3, 4. Macrovascular complications stem from accelerated cardiovascular diseases (CVDs), culminating in myocardial infarction and strokes 5. Given that diabetes significantly contributes to blindness, kidney failure, heart attacks, and limb amputation, the imperative to address diabetes and its associated vasculopathy is substantial 2, 3.

While the primary treatment for diabetes focuses on normalizing blood glucose levels 6, both experimental and clinical studies have demonstrated that glucose normalization alone does not suffice in preventing diabetic complications. This phenomenon, termed hyperglycemic memory (HGM) or the legacy effect, underscores the impact of persistent hyperglycemic stress 7-10. Effectively treating diabetes necessitates not only the normalization of hyperglycemia but also the prevention of hyperglycemic memory-induced diabetic complications. Therefore, understanding the pathological mechanism(s) underlying microvascular and macrovascular diabetic complications and identifying therapeutic targets for these complications are pivotal for developing comprehensive treatment strategies.

Transglutaminase 2 (tissue transglutaminase; TGase2) belongs to the transglutaminase family and catalyzes Ca^2+^-dependent protein crosslinking through the transamidation of glutamine residues to lysine residues 11. Ubiquitously expressed, TGase2 is a multifunctional enzyme, acting as a transamidase, serine/threonine kinase, protein disulfide isomerase, and GTPase 11-14. Additionally, TGase2 demonstrates non-enzymatic functions through interactions with extracellular proteins, such as fibronectin, integrins, and syndecans, promoting matrix stabilization, which contributes to fibrosis in the kidney, liver, heart, and lung 11, 15.

Participating in various physiological processes, TGase2 influences apoptosis, inflammation, epithelial-mesenchymal transition, fibrogenic reactions, and mitochondrial dysfunction through post-translational modifications of several substrate proteins, including collagen, gluten, tau, α-synuclein, and β-crystallin 11, 13, 15-17. Given its multifaceted roles, TGase2 is implicated in the pathogenesis of diverse diseases, including celiac disease 18, neurodegenerative diseases 13, 19, cancers 16, 20, fibrosis 17, 21, inflammatory diseases 22, and heart failure 23.

Recently, TGase2 has emerged as a key enzyme in the pathogenesis and therapeutic investigation of diabetic vascular complications, including DR, DN, DP, and CVD 4, 24-27. This review focuses on TGase2's multifaceted role in diabetic complications, exploring its potential as a therapeutic target and discussing the emerging landscape of TGase2 inhibitors for addressing these intricate vascular complications.

## 2. Regulation and functions of TGase2 transamidase and kinase activities

Among the four distinct enzymatic activities of TGase2, the transamidase and kinase activities are most likely to be involved in the pathogenesis of diabetic complications 24, 28-31. Consequently, this review focuses on the regulation and functions of these two enzymatic activities. The intricate interplay between these activities is influenced by various regulators, including thiol compounds, divalent metal cations, and phosphorylation 11, 14, 32 (**Table 1**). Additionally, they share several common proteins as substrates, such as nuclear factor-κB (NF-κB), retinoblastoma protein (pRb), E-cadherin, and p53 11, 30, 33 (**Table 2**). Thus, to grasp the role of TGase2 in the pathogenesis of diabetic complications, a thorough understanding of the regulatory mechanisms governing TGase2 transamidase and kinase activities, along with their respective target proteins, becomes imperative.

### 2.1. TGase2 transamidase activity

#### 2.1.1. Regulation of TGase2 transamidase activity

**Figure 1:**
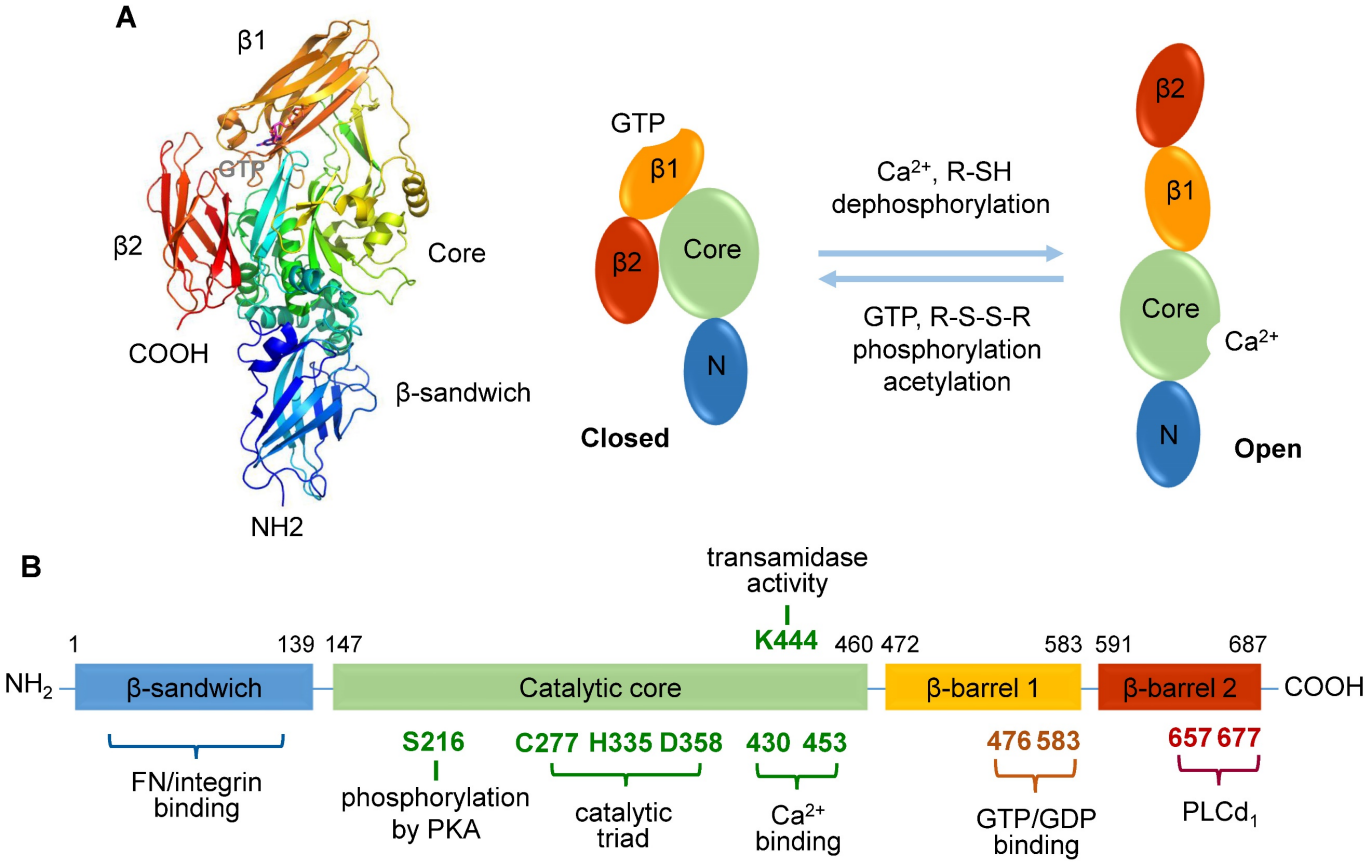
** Human transglutaminase 2 (TGase2) open and closed structures and amino acid regulation map. A.** TGase2 exhibits open and closed structural formations. The closed TGase2 structure was prepared with Pymol 3.0 using the PBD file 4PYG. **B.** A comprehensive amino acid map illustrating the regulatory mechanisms of TGase2 activity. β1; β-barrel 1 domain; β2, β-barrel 2 domain; Core, core catalytic domain; N, N-terminal β-sandwich domain; FN, fibronectin; PKA, protein kinase A; PLCδ1, phospholipase C δ1.

TGase2 comprises four distinct domains: an N-terminal β-sandwich domain, a catalytic core domain, and two C-terminal β-barrel domains 34, 35 (**Figure 1A**). While the N-terminal β-sandwich domain lacks catalytic activity, it is recognized for its binding affinity to fibronectin and integrin 35. The catalytic core domain, housing a catalytic triad (Cys277, His335, and Asp358), possesses five calcium-binding sites 36, and β-barrel 1 features a GTP/GDP-binding site **(Figure 1B)**. Three-dimensional structural analysis has revealed the reciprocal regulation of transamidase activity by Ca^2+^, nucleotides, and various regulators 11. TGase2 bound with GDP adopts a closed conformation, negatively regulating its transamidase activity. In contrast, the transamidase activity is induced through Ca^2+^ binding, resulting in an open conformation in which the transamidase active site is exposed to substrates.

In addition to Ca^2+^ and nucleotides, the transamidase activity of TGase2 is subject to regulation by amine compounds, nitric oxide, and thiol compounds (**Table 1**) 11, 14. Amine compounds, such as putrescine, monodansylcadaverine (MDC), 5-(biotinamido)pentylamine (BAPA), spermidine, and histamine, act as amine donors to inhibit transamidase activity by competing with natural substrates 17, 37, 38. Nitric oxide also inhibits the transamidase activity through S-nitrosylation of the cysteine residue in the active site 39. Thiol compounds, including glutathione (GSH), cystamine, and cysteamine, potentially regulate transamidase activity by affecting the redox state of TGase2 cysteine residues, particularly the catalytic core residue (Cys277) and three cysteine residues (Cys230, Cys370, and Cys371) 14, 25, 40.

Several amino acids in TGase2 play a role in regulating transamidase activity 41 **(Figure 1B)**. Catalytic Cys277 is essential for transamidase activity, and this function is further influenced by disulfide bond formation among three cysteine residues (Cys230, Cys370, and Cys371) 40, 41. A site-directed mutagenesis study identified Lys444 in the catalytic domain as significant for transamidase activity 41. Interestingly, the phosphorylation levels of TGase2 also regulate its transamidase activity 14. Dephosphorylation of TGase2 with alkaline phosphatase enhances transamidase activity, whereas phosphorylation using the catalytic subunit of protein kinase A (PKA) inhibits this enzyme 14. However, the role of acetylation in the regulation of transamidase activity remains controversial 14, 42.

#### 2.1.2. Functions of TGase2 transamidase activity

TGase2 transamidase plays a pivotal role in the regulation of various proteins, including RhoA 43, NF-κB 44, glyceraldehyde 3-phosphate dehydrogenase (GAPDH) 45, pRb 46, and the oncoprotein p53 47 (**Table 2**). Retinoic acid-induced activation of the transamidase leads to increased transamidation of RhoA at Gln63, a process inhibited by MDC 43, 48. Transamidated RhoA, acting as a constitutively active G-protein, enhances its binding to RhoA-associated kinase-2, fostering the formation of stress fibers and focal adhesion complexes. This suggests the involvement of transamidase activity in cell differentiation, including that of human leukemia cells and neurons 43, 48, 49. The transamidase also activates NF-κB by inducing the polymerization of I-κB, resulting in subsequent NF-κB dissociation and translocation into the nucleus. This activation is capable of upregulating inflammatory genes such as inducible NO synthase and tumor necrosis factor α 44.

Furthermore, the transamidase inhibits GAPDH, a glycolytic enzyme, through crosslinking lysine residues in the C-terminal region of GAPDH with polyglutamine repeats. This interference leads to disruptions in energy metabolism 45, 50. Inhibition of GAPDH activity elevates the levels of glycolysis intermediates or their metabolites, resulting in the activation of pathological vascular complications, including diabetic complications 3. Modification of pRb by the transamidase protects from apoptosis by inhibiting caspase-induced degradation of polymerized pRb 46, 51. Integrative proteomic profiling of the transamidase activity using protein arrays has identified several potential substrates, including osteopontin and globular actin 33, suggesting potential roles of the transamidase in cytoskeleton and bone remodeling. The TGase2 transamidase activity is also implicated in tumorigenicity 47 and pulmonary fibrosis 21.

### 2.2. TGase2 kinase activity

#### 2.2.1. Regulation of TGase2 kinase activity

The regulatory mechanism of TGase2 kinase activity is less understood compared to the transamidase activity, primarily due to limitations in suitable assays for determining *in situ* and *in vivo* kinase activity 30. Determination of kinase activity has traditionally involved detecting ATP incorporation into substrate proteins using radioactive isotopes 52, 53 or antibodies against phosphoamino acids 52. However, these methods have drawbacks, such as the use of hazardous radioactive probes or relatively low affinities for phosphorylated substrates 30. To overcome these limits, a well-type array-based kinase activity assay has been introduced using Pro-Q Diamond phosphoprotein stain 30.

Investigations into the regulation mechanism of TGase2 kinase have utilized on-chip kinase activity assays 14, 32. The kinase is regulated by thiol compounds, divalent metal cations, phosphorylation, and acetylation 14, 32 (**Table 1**). The kinase activity assay, employing a cysteine-modified insulin-like growth factor-binding protein-3 (IGFBP-3)-derived peptide, revealed that thiol compounds, such as 5,5′-dithio-bis-(2-nitrobenzoic acid), S-nitroso-N-acetylpenicillamine, dithiothreitol, GSH, and mercaptoethanol, inhibit the kinase (**Table 1**). Thus, the modification of TGase2 cysteine residues may play a critical role in regulating the TGase2 kinase 32. Notably, cystamine, a disulfide compound with amine moieties at both ends, enhances the kinase activity 14, even though this disulfide compound inhibits TGase2-induced phosphorylation of IGFBP-3 54. Divalent metal cations also play a role in the regulation of TGase2 kinase, as on-chip activity assays demonstrate kinase inhibition by Cu^2+^, Mn^2+^, Ni^2+^, and Zn^2+^, but not by Ca^2+^ 32. Furthermore, phosphorylation and acetylation of TGase2 enhance the kinase activity, with acetylation of TGase2 not affecting transamidase activity 14.

#### 2.2.2. Functions of TGase2 kinase activity

TGase2 can phosphorylate serine and threonine, but not tyrosine, residues of substrate proteins 55. The intrinsic kinase activity of TGase2 was revealed by Mishra and Murphy 54, who demonstrated the phosphorylation of IGFBP-3 by TGase2 on breast cancer cell membranes. TGase2 undergoes phosphorylation at Ser216 by PKA, resulting in activation of NF-κB and protein kinase B, downregulation of phosphatase and tensin homolog (PTEN) 56, phosphorylation of pRb 52, and enhanced TGase2 binding to 14-3-3 57 **(Table 2)**. TGase2 kinase further phosphorylates p53 58 and histone proteins 53, indicating its role in apoptosis regulation and chromatin structure and function, respectively. p53 is a key tumor suppressor protein that has multiple biological functions, including DNA damage repair, cell cycle arrest, apoptosis, and senescence 59. Additionally, the kinase phosphorylates β-catenin at Tyr654 through a c-Src-dependent mechanism, leading to the proliferation of ovarian cancer cells. However, the precise mechanism by which TGase2 activates c-Src remains unclear 31.

An on-chip kinase activity assay using Pro-Q Diamond stain, suitable for screening the kinase substrates, was employed to investigate potential substrates of TGase2 kinase 30. This high-throughput activity assay revealed several kinase substrate proteins based on substrate affinity (K_m_), including glucose 6-phosphate dehydrogenase, cytochrome C, calmodulin, and S100 calcium-binding protein A7 in the cytosol. This suggests potential roles of TGase2 kinase in regulating oxidative stress 60, apoptosis 61, and intracellular Ca^2+^ signaling events 62. TGase2 kinase also phosphorylates E-cadherin in the plasma membrane and matrix metalloproteinase-3 (MMP-3) in the extracellular matrix, indicating its potential involvement in the breakdown of extracellular matrix and cancer metastasis 63, 64.

## 3. TGase2 as a therapeutic target for diabetic complications

Oxidative stress plays a pivotal role in the pathogenesis of diabetic complications, leading to progressive vascular damage and dysfunction 1, 65, 66. These vascular complications are associated with various pathological pathways, including increased polyol pathway flux, elevated hexosamine biosynthesis, activation of protein kinase C, and augmented formation of advanced glycation end products 3, 8. These pathways are triggered by hyperglycemia-induced inhibition of GAPDH through poly ADP-ribosylation 67. Recent reports emphasize that hyperglycemia-induced generation of intracellular reactive oxygen species (ROS) plays a crucial role in activating TGase2, contributing to diabetic microvascular and macrovascular complications 3, 24, 27. Importantly, a vicious cycle exists between hyperglycemia-induced ROS generation and TGase2 activation, which significantly contributes to HGM-induced endothelial dysfunction 28, 65.

This section delves into the pivotal role of TGase2 in diabetic complications, specifically DR, DN, DP, and CVD. Furthermore, we explore the role of TGase2 in HGM, a significant phenomenon in the development and progression of diabetic complications in both type 1 and type 2 diabetes 28, 65. It is important to note that this review excludes discussions on diabetic neuropathy, impaired wound healing, and stroke due to the unclear role of TGase2 in the pathogenesis of these complications.

### 3.1. Diabetic retinopathy

DR is the most common diabetic microvascular complication and remains the leading cause of blindness in working-age populations 68, 69. This metabolic disease progresses from early non-proliferative DR to late proliferative DR 70, 71. DR is influenced by various risk factors such as poor glucose control, diabetes duration, hypertension, and plasma glucose fluctuations 72. Non-proliferative DR represents the early stages, characterized by thickening of the basement membrane, pericyte loss, formation of acellular capillaries, microaneurysms, and microvascular leakage 5, 70. Proliferative DR, on the other hand, features pathological neovascularization and eventual diabetic macular edema, contributing to vision loss 73, 74.

**Figure 2:**
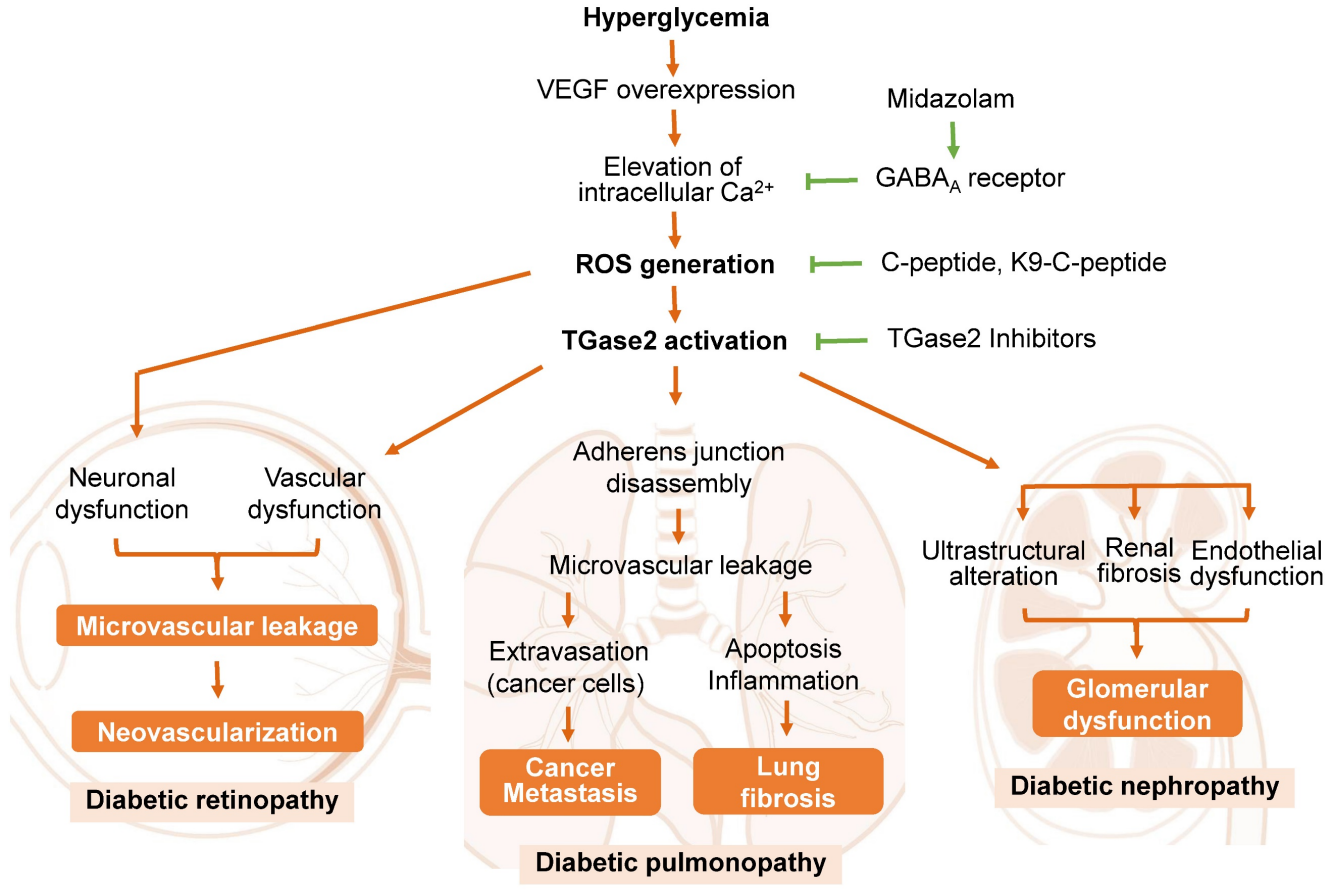
** The role of transglutaminase 2 (TGase2) in the pathogenesis of diabetic retinopathy, pulmonopathy, and nephropathy.** Reactive oxygen species (ROS)-mediated activation of TGase2 contributes to hyperglycemia-induced microvascular leakage and neovascularization in the retina, cancer metastasis and fibrosis in the lung, and glomerular dysfunction in the kidney. GABA_A_, γ-aminobutyric acid type A; K9-C-peptide, human C-peptide conjugated with nine repeats of lysine-containing elastin-like polypeptide; VEGF, vascular endothelial growth factor.

A predominant cause of microvascular leakage in the diabetic retina is hyperglycemia-induced overexpression of vascular endothelial growth factor (VEGF) 24, 75, 76 **(Figure 2)**. Elevated VEGF levels in the retina activate TGase2 through sequential elevation of intracellular Ca^2+^ and ROS, leading to microvascular leakage through stress fiber formation and disassembly of adherens junctions 24, 76. Thus, ROS-mediated activation of TGase2 plays a key role in VEGF-induced retinal vascular leakage, positioning TGase2 as a potential therapeutic target for DR treatment in several reports 3, 25, 28, 71, 77.

Proinsulin C-peptide, administered systemically or intravitreally, alleviates VEGF-induced retinal microvascular leakage by inhibiting ROS-mediated activation of TGase2, subsequently preventing stress fiber formation and vascular endothelial (VE)-cadherin disassembly in diabetic mice 24, 71, 76. Cysteamine, an aminothiol derived from coenzyme A degradation, attenuates vascular leakage by inhibiting VEGF-induced activation of TGase2 and VE-cadherin disruption in diabetic retinas 25. The benzodiazepine anesthetic midazolam reduces hyperglycemia-induced vascular leakage by inhibiting VEGF-induced elevation of intracellular Ca^2+^ and subsequent ROS generation and TGase2 activation through the GABA_A_ receptor in the retinas of diabetic mice 77. While TGase2 plays a crucial role in VEGF-induced retinal vascular dysfunction, it is not associated with diabetes-induced retinal neurodegeneration 71. Thus, TGase2 has emerged as a significant enzyme in the pathogenesis of retinal vascular dysfunction in diabetic retinas and represents a promising therapeutic target for DR.

### 3.2. Diabetic pulmonopathy

DP is a newly recognized microvascular complication of diabetes 3, 78. While diabetes is well-established as a systemic disease with chronic oxidative stress and inflammation affecting various organs, such as the eyes, kidneys, nerves, skin, and the vascular system 79, 80, the pulmonary complications have been comparatively overlooked. This disregard is attributed to the lung's significant physiological reserve and the presence of subclinical pulmonary abnormalities in diabetic patients 78, 80, 81. Nevertheless, recent clinical and experimental investigations highlight the lung as a potential target organ affected by diabetes 78, 82, 83. The intricate alveolar-capillary network and the abundance of collagen and elastin in the lung render it susceptible to diabetic microvascular damage 78, 80, 84. Type 1 and type 2 diabetes patients exhibit respiratory abnormalities, including reduced diffusing capacity, lung volume, control of ventilation, and elastic recoil 81, 85. Clinical studies also suggest associations between diabetes and conditions such as asthma, idiopathic fibrosis, chronic obstructive pulmonary disease, and hypertension in the lungs of diabetic patients 80, 82, 83, 86.

Idiopathic pulmonary fibrosis, characterized by chronic, progressive fibrotic lung disease with high mortality and limited therapeutic options 84, 85, shows abnormal extracellular matrix accumulation in the lung destroying alveolar architecture, which results in pulmonary dysfunction and respiratory failure 87. Emerging research indicates a potential link between idiopathic pulmonary fibrosis and diabetes 80, 82-85, with epidemiological research identifying diabetes as an independent risk factor for idiopathic pulmonary fibrosis 88.

The underlying mechanisms of DP have been investigated in rodent models of type 1 diabetes 28, 78, 79. In diabetic rats, hyperglycemia induces fibrotic changes in the lung by activating TGF-β-mediated epithelial-mesenchymal transition 79. Notably, recent findings by Ha and colleagues 4 highlight that chronic hyperglycemia promotes vascular leakage in the lungs through VEGF-induced ROS generation and subsequent TGase2 activation, leading to melanoma cell metastasis in diabetic mice **(Figure 2)**. The hyperglycemia-induced vascular leakage and melanoma cell metastasis were mitigated by inhibiting TGase2 through systemic supplementation of proinsulin C-peptide 4, insulin 26, or midazolam 89.

TGase2 is also implicated in hyperglycemia-induced inflammation and apoptosis, contributing to pulmonary fibrosis in the diabetic lung. This pathological process was ameliorated through long-term systemic supplementation of human C-peptide, achieved by subcutaneous implantation of a thermosensitive biopolymer-conjugated C-peptide (K9-C-peptide) depot 28, 78. The involvement of TGase2 in pulmonary fibrosis was further confirmed in a mouse model of bleomycin-induced pulmonary fibrosis 21. Consequently, TGase2 is a promising therapeutic target for addressing multiple facets of DP, including microvascular leakage, inflammation, metastasis, and idiopathic fibrosis.

### 3.3. Diabetic nephropathy

DN, a significant microvascular complication affecting approximately 40% of diabetic patients, is the primary cause of chronic kidney disease leading to end-stage renal disease in developed countries 90, 91. Clinically, DN manifests as albuminuria, a progressive decrease in glomerular filtration rate, and elevated blood pressure 92. Pathologically, this chronic kidney disease is characterized by glomerulosclerosis, tubulointerstitial fibrosis, thickening of glomerular and tubular membranes, and vascular dysfunction 93, 94. While the prevalence of diabetic kidney disease has surged over the past decades, currently available treatments are limited to those preventing or delaying disease progression 90.

Microalbuminuria, widely acknowledged as the initial clinical sign of DN, is induced by the disruption of the glomerular filtration barrier due to glomerular basement membrane thickening, foot process effacement of podocytes, and microvascular dysfunction 93. Notably, microvascular dysfunction, arising from VEGF overexpression and subsequent vascular integrity disruption, plays a pivotal role in the pathogenesis of diabetic microvascular complications in the retina and lungs 4, 76, 95. In the kidney, glomerular endothelial dysfunction has been implicated in the pathogenesis of diabetic kidney disease 95, 96. However, the underlying mechanism by which hyperglycemia induces alterations in glomerular endothelial permeability and subsequent microalbuminuria remains unclear. Recent studies have highlighted the role of TGase2 in glomerular endothelial dysfunction and renal fibrosis in diabetic animal models 27, 93, 97** (Figure 2)**.

In the renal cortex of type 1 diabetic mice, hyperglycemia activates the transamidase activity of TGase2 and induces apoptosis, both of which are suppressed by systemic supplementation of human C-peptide 27. TGase2's involvement has been reported in mouse models of renal fibrosis induced by unilateral ureteral obstruction or streptozotocin 93, 98. TGase2 contributes to interstitial renal fibrosis through TGF-β activation and cell infiltration in unilateral ureteral obstruction mouse models 98. Additionally, TGase2 plays a role in hyperglycemia-induced pathological alterations of glomerular ultrastructure and renal fibrosis, with midazolam attenuating glomerular endothelial dysfunction in the kidneys of diabetic mice 93. Despite the formidable challenge of elucidating the pathological mechanisms of DN, TGase2 is a potential therapeutic target for its treatment.

### 3.4. Cardiovascular disease

CVD, the most prevalent diabetic complication, is the leading cause of death in patients with either type 1 or type 2 diabetes 3, 99, 100. CVD encompasses a group of disorders affecting the heart and blood vessels, including premature atherosclerosis leading to myocardial infarction, stroke, and compromised cardiac function 5, 101. Its high prevalence in type 1 diabetes patients significantly impairs life expectancy 100, 102, with chronic kidney disease and cardiac autonomic neuropathy being associated with its progression in these patients 100, 103. In type 2 diabetes, kidney disease remains a major risk factor for CVD, accompanied by other CVD risk factors, including dyslipidemia, poor glycemic control, and persistent high blood pressure 5, 101.

Several reports propose the involvement of TGase2 in the pathogenesis of CVDs, including atherosclerosis, myocardial fibrosis, and associated heart diseases 23, 104-106. However, studies specifically addressing the role of TGase2 in diabetes-associated CVDs are limited. Investigations into hyperglycemia-induced progression to CVD in the aorta of type 1 diabetic mice 27, 65 indicate that hyperglycemia stimulates transamidating activity and endothelial cell apoptosis in the aortic endothelium, processes inhibited by systemic supplementation of human C-peptide 27. Administering human C-peptide through osmotic pumps protects endothelial cells from hyperglycemia-induced apoptosis by blocking ROS-mediated activation of TGase2. This ROS generation and TGase2 activation form a vicious cycle implicated in hyperglycemia-induced vascular dysfunction, including the expression of inflammatory adhesion molecules and apoptosis 65. Nevertheless, further research is essential to comprehensively understand the role of TGase2 in the pathogenesis of diabetes-associated CVD.

### 3.5. Hyperglycemic memory

HGM, signifying the persistence of hyperglycemic stress post-glucose normalization, is a crucial factor in the pathogenesis and progression of both diabetic microvascular and macrovascular diseases in type 1 and type 2 diabetes 3, 10, 28. Engerman and Kern's seminal work 7 reveals that intensive glycemic control fails to arrest the progression of DR in diabetic dogs initially subjected to poor glycemic control, characterized by capillary aneurysm, acellular capillaries, and pericyte loss. Large-scale clinical studies, such as the Diabetes Control and Complications Trial (DCCT)-Epidemiology of Diabetes Interventions and Complications (EDIC) study in type 1 diabetes patients 10, 107 and the UK Prospective Diabetes Study (UKPDS) in type 2 diabetes patients 108, 109, demonstrate that initial tight glycemic control diminishes the incidence of diabetic complications. Conversely, initial poor glycemic control can lead to the long-term development of diabetic complications 110.

To unravel the underlying mechanism of persistent hyperglycemic stress and understand the pathophysiology of diabetic complications, diabetic animal models have been employed 9, 65, 111, 112. Persistent oxidative stress plays a central role in the long-lasting vascular complications induced by HGM 8, 65, 110, 113. Hyperglycemia induces sustained upregulation of pro-oxidant enzymes, namely protein kinase C β and p47phox, a NADPH oxidase subunit, post-glucose normalization in the retina of diabetic rats 112. The prolonged activation of p66^Shc^ by protein kinase C βII maintains ROS generation after normoglycemia, resulting in vascular apoptosis in the mouse aorta 110. p66^Shc^, a 66 kDa Src homology/collagen adaptor protein, is a key regulator of mitochondrial function, oxidative stress, and apoptosis 114.

**Figure 3:**
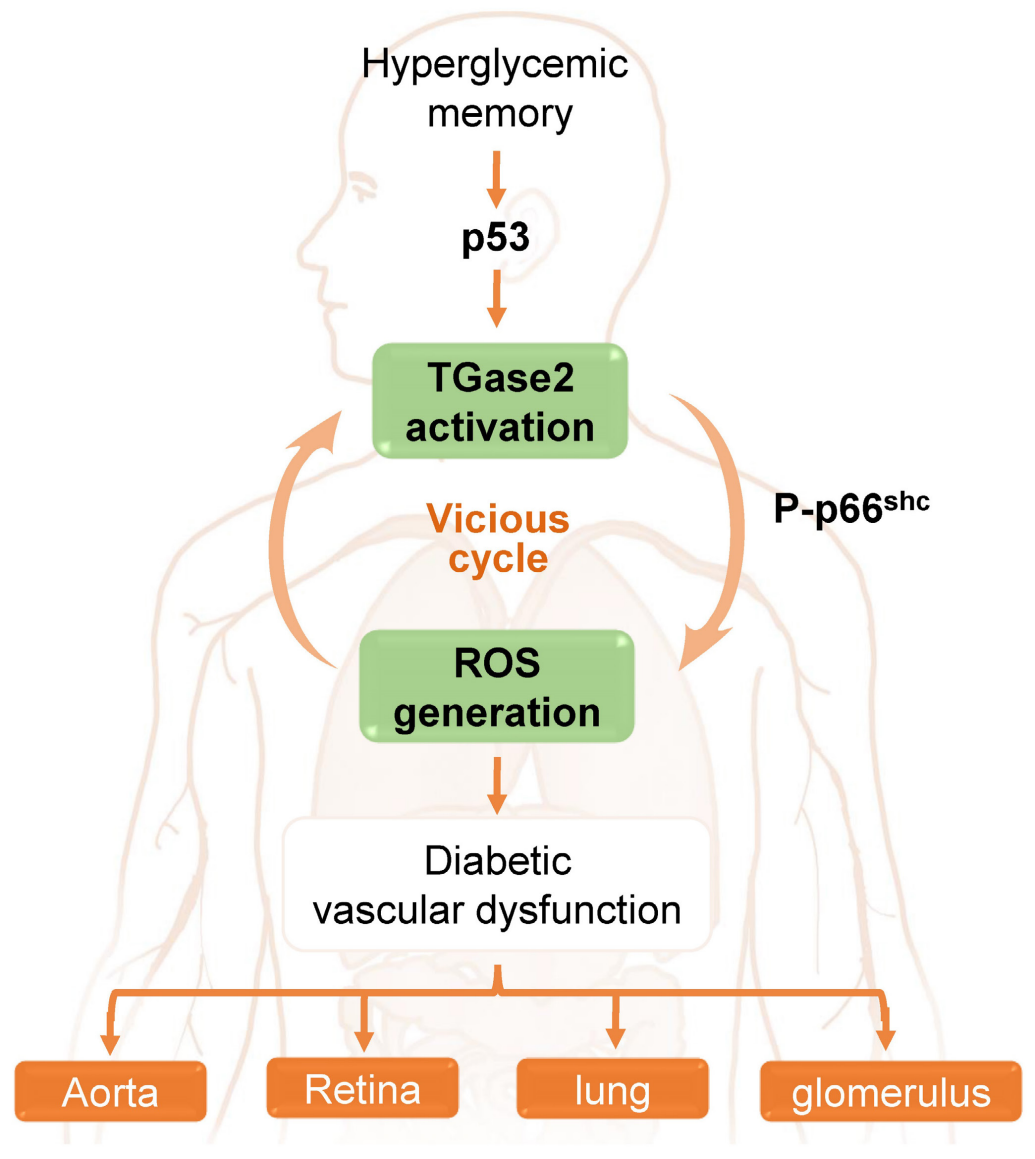
The role of a transglutaminase 2 (TGase2)-reactive oxygen species (ROS) vicious cycle in hyperglycemic memory-induced vascular dysfunction in the aorta, retina, lung, and glomerulus.

In the aorta of diabetic mice, Ha and colleagues 65 elucidated that hyperglycemia induces sustained ROS generation and TGase2 activation post-return to normoglycemia, initiating a detrimental cycle. This ROS-TGase2 cycle, regulated by the mitochondrial adaptor p66^Shc^ and p53, is implicated in the sustained expression of inflammatory adhesion molecules and apoptosis in the aortic endothelium 65, 111. Furthermore, this vicious cycle is associated with HGM-induced complications in the retina, including vascular leakage and neurodegeneration, as well as in the lungs, where it leads to vascular leakage and fibrosis. Additionally, it contributes to other complications such as glomerular adherens junction disruption and vascular leakage in diabetic mice 28. Thus, TGase2, activated by persistent oxidative stress and associated with p66^Shc^ and p53, assumes a pivotal role in HGM-induced vascular complications in the aorta, retina, lung, and kidney **(Figure 3)**.

## 4. Inhibition of TGase2 as a potential therapeutic strategy for diabetic complications

TGase2 plays a pivotal role in the pathogenesis of diabetic complications, such as DR, DN, DP, CVD, and HGM. These microvascular and macrovascular complications, stemming from chronic hyperglycemia and hyperglycemia memory, can be addressed through the direct or indirect inhibition of TGase2. Direct inhibition encompasses the use of competitive, reversible, and irreversible inhibitors that affect substrate binding to the active site of TGase2. Indirect inhibition by G-protein-coupled receptor agonists, such as C-peptide, C-peptide conjugates, and midazolam, regulate TGase2 by binding to its receptors.

### 4.1. Direct inhibition

#### 4.1.1. Competitive inhibitors: monoamines

Competitive inhibitors, which consist of biogenic monoamines and polyamines, regulate TGase2 activity by competing with its natural substrates rather than preventing the enzyme activity through covalent modification or allosteric regulation during the transamidation reaction, leading to the formation of isopeptide bonds between the natural glutamine substrates and the inhibitors 37. Amine inhibitors have been intensively used in cellular and organismal studies due to their commercial availability, stability, and relative non-toxicity 37, 97. Uteroglobin, a protein substrate of TGase2, has also been employed as a competitive TGase2 inhibitor in murine experimental crescentic glomerulonephritis 115.

Monoamine inhibitors, including catecholamines and amino acid derivatives, are characterized by a primary amine linked to alkyl chains 37, 97, 116. Among the biogenic monoamines, cysteamine, MDC, and dopamine have demonstrated efficacy against diabetic complications **(Table 3)**. Cysteamine, also known as β-mercaptoethylamine, an aminothiol with a primary amine group and a sulfhydryl group, is endogenously derived from coenzyme A degradation and metabolized into taurine 25, 117. Cysteamine exhibits therapeutic potential for DR and vascular leakage-associated diseases 25. Additionally, the aminothiol is beneficial in conditions such as cystinosis 117, nonalcoholic fatty liver disease 118, neurodegenerative diseases like Alzheimer's, Huntington's, and Parkinson's diseases 119, 120, and postinflammatory hyperpigmentation 121. There is a recent report from three clinical trial programs 122, suggesting long-term clinical benefits of delayed-release cysteamine bitartrate capsules in patients with nephropathic cystinosis.

MDC has been used to investigate the role of TGase2 in the pathogenesis of diabetic complications 4, 24, 93. MDC inhibits VEGF-induced stress fiber formation, adherens junction disassembly, and subsequent endothelial permeability in human retinal endothelial cells, attenuating hyperglycemia-induced microvascular leakage in the retinas of diabetic mice 24, 77. Moreover, MDC suppresses VEGF or high glucose-induced VE-cadherin disassembly and endothelial permeability in human pulmonary microvascular endothelial cells 4 or human glomerular microvascular endothelial cells 93, indicating its benefit against DR, DP, and DN.

Dopamine, a neurotransmitter in the nervous system, has beneficial effects against DR 113. In the retina, dopamine, the most abundant catecholamine, is released from dopaminergic neurons identified as dopaminergic amacrine cells and interplexiform cells 123. In type 1 diabetic mice, dopamine deficiency is associated with early visual dysfunction, which is improved by intraperitoneal treatment with dopamine receptor agonists or the dopamine precursor levodopa 124, 125. Furthermore, in the retinas of type 1 diabetic mice, HGM-induced persistent stresses, including oxidative stress, mitochondrial membrane potential collapse and fission, and adherens junction disassembly leading to vascular leakage, are attenuated by intravitreal injection of levodopa 113.

Moreover, BAPA holds therapeutic promise for diabetic complications. This is attributed to its extensive utilization as a pseudosubstrate in *in vitro* 4, 33, 126, 127, *in situ* 27, 28, 128, 129, and *in vivo* 4, 24, 28 TGase activity assays.

#### 4.1.2. Competitive inhibitors: polyamines

There exist various biogenic polyamine inhibitors of TGase2, including cystamine, putrescine, spermine, and spermidine, that compete with substrate proteins in the transamidation reaction **(Table 3)**. Cystamine is a frequently utilized polyamine for investigating TGase2's function in the pathogenesis of diabetic vascular dysfunctions in multiple tissues, including the aorta 65, eye 24, 71, lung 4, 78, and kidney 89, 93. Distinguished by its intricate regulatory mechanisms compared to other competitive inhibitors 37, 97, cystamine is reduced into cysteamine due to the high molar ratio of GSH to GSSG (100:1) in resting cells 130. Cysteamine, the reduced form of cystamine, functions as a competitive amine inhibitor. It can irreversibly bind to the catalytic cysteine residues of TGase2, forming disulfide bonds 37. Additionally, cystamine elevates intracellular GSH levels 131, influencing oxidative stress-associated pathophysiological responses 97. Furthermore, cystamine increases brain and serum levels of the neuronal survival factor brain-derived neurotrophic factor and has therapeutic potential for neurodegenerative diseases and schizophrenia 132.

Polyamines derived from ornithine, such as putrescine, spermidine, and spermine, serve as TGase2 substrates 133. Ornithine is converted into putrescine by ornithine decarboxylase, followed by sequential conversion into spermidine and spermine by spermidine synthase and spermine synthase, respectively 134. These ornithine-derived polyamines play a crucial role in normal β-cell functions, influencing intracellular Ca^2+^ levels and inflammation. Consequently, alterations in polyamine levels are implicated in the pathogenesis of diabetes 135. Due to their positive charge at physiological pH, these polyamines strongly interact with negatively charged molecules like DNA and RNA, participating in DNA replication, gene expression, and mRNA translation 135. Elevated serum levels of putrescine and spermine in type 2 diabetic patients suggest the potential involvement of polyamine metabolism in the pathogenesis of type 2 diabetes 136. However, the precise association between the roles of putrescine, spermidine, and spermine in normal β-cell functions and the pathogenesis of type 2 diabetes remains unclear, particularly regarding their impact on TGase2 inhibition.

#### 4.1.3. Irreversible TGase2 inhibitors

Several irreversible TGase2 inhibitors have been reported by various research groups, including chloromethyl ketones, dihydroisoxazole derivatives, Michael acceptors, and gluten peptide analogs 37, 116, 137 **(Table 3)**. Irreversible TGase2 inhibitors, commonly termed suicide inhibitors, exert their effect by covalently modifying the enzyme, primarily at the active site, thus hindering substrate binding to the active site 37.

Iodoacetamide, an early irreversible inhibitor, was employed to inhibit guinea pig TGase2 by forming a thioester bond with the cysteine residue of the enzyme's active site 138. A series of chloromethyl ketones, synthesized based on a peptidic (carbobenzyloxy-phenylalanine) scaffold, underwent testing for reactivity toward glutathione 139. Dihydroisoxazole derivatives, derived from the natural product acivicin, represent a well-studied class of TGase2 irreversible inhibitors. Exhibiting various biological activities 140, including anti-inflammatory 141, anticancer 142-144, and antioxidant effects 145, dihydroisoxazoles hold potential for treating diabetic complications. NTU compounds, such as NTU281 (N-benzyloxycarbonyl-L-phenylalanyl-6-dimethylsulfonium-5-oxo-L-norleucine) and NTU283 (1,dimethyl-2[(oxopropyl)thio]imidazolium), bind irreversibly to the TGase2 catalytic cysteine residues 97, 146. NTU281 showed beneficial effects against hyperglycemia-induced glomerular dysfunction in diabetic rats 147, 148.

Michael acceptors, as reported by Keillor and colleagues 116, belong to a family of irreversible TGase2 inhibitors with the carbobenzyloxy-phenylalanine scaffold and acrylamide warhead. Zedira also documented several peptidomimetic TGase2 inhibitors featuring an α,β-unsaturated ester, designed for treating celiac disease and TGase2-associated ailments 137. Gluten is a complex protein mixture with immunogenic peptide sequences triggering autoimmune responses in patients with celiac disease 149. Gluten peptides have been utilized as peptidomimetic irreversible inhibitors due to its high affinity toward TGase2. The glutamine in these peptides has been substituted with acivicin or 6-diazo-5-oxo-norleucine (DON) to enhance the inhibitory potency 150, 151, while the DON peptide was shown to be more potent in inhibiting TGase2 than the acivicin analogue 150. Acetylsalicylic acid, commonly known as aspirin, impacts the transamidation or kinase activity of TGase2 through lysine residue acetylation 14, 42. However, its effect on diabetic complications remains poorly understood.

#### 4-1-4. Reversible inhibitors

Reversible TGase2 inhibitors curtail enzyme activity by impeding substrate access to the active site without instigating covalent modifications 37. Allosteric regulation of TGase2 is achieved through GTP analogs and thieno[2,3-d]pyrimidin-4-one acylhydrazides (LDN27219) 37, 116. Nonhydrolyzable GTP analogs, such as GTPγS and GMP-PCP, reversibly inhibit TGase2 129, 152. LDN27219, categorized as a reversible inhibitor, exhibits slow binding to the GTP site of TGase2, inducing a closed conformation and inhibiting transamidase activity 153. In arteries, LDN 27219 shows promise in lowering blood pressure and enhancing endothelium-dependent vasorelaxation, suggesting its potential in addressing vascular dysfunction 154 **(Table 3)**.

TGase2-specific siRNA has emerged as a potential therapeutic avenue for diabetic complications. Intravitreal injection of TGase2 siRNA attenuates hyperglycemia-induced retinal vascular leakage in type 1 diabetic mice 24. Moreover, TGase2 siRNA inhibits VEGF and high glucose-induced oxidative stress, VE-cadherin disassembly, and endothelial permeability in human retinal endothelial cells, human pulmonary microvascular endothelial cells, and human glomerular microvascular endothelial cells 4, 24, 93, underscoring its potential role in treating DR, DP, and DN **(Table 3)**.

Various other reversible TGase2 inhibitors have been reported, including trans-cinnamoyl derivatives, cinnamoyl benzotriazolyl amides and azachalcones 155, ZM39923 and ZM449829 156, acylideneoxoindoles 157, and quinoxaline derivatives 158. However, the precise roles of these inhibitors in diabetic complications remain unclear. Further investigations are warranted to elucidate their impact on mitigating the effects of diabetes-related pathologies.

### 4.2. Indirect inhibition

#### 4.2.1. Proinsulin C-peptide

Human proinsulin C-peptide, a 31-amino acid peptide, is released from pancreatic β-cells into the portal circulation in equimolar concentrations with insulin 159. Discovered in 1967, C-peptide serves as an indicator of β-cell function. Since the early 1990s, C-peptide has been recognized for its potential benefits in treating diabetic microvascular and macrovascular complications 3, 160 **(Table 4)**.

C-peptide exhibits a reduction in ROS generation by attenuating NADPH oxidase activation in human aortic endothelial cells 161, potentially mediated through binding to G protein-coupled receptor 146 162. In various human endothelial cells (retina, lung, aorta, and umbilical vein), C-peptide inhibits ROS-mediated TGase2 activation, preventing high glucose- or VEGF-induced VE-cadherin disassembly and endothelial permeability 4, 24, 27, 65. C-peptide replacement ameliorates hyperglycemia-induced vascular dysfunction in the aorta 27, 65, heart 27, renal cortex 27, retina 24, and lung 4, 78, highlighting its beneficial impact on diabetic microvascular and macrovascular complications. Additionally, systemic C-peptide supplementation is effective in preventing impaired wound healing by stimulating angiogenesis and inhibiting inflammation 163. Furthermore, C-peptide improves renal dysfunction, such as hyperfiltration and albumin excretion, and alleviates autonomic and sensory nerve dysfunction in patients and animal models with type 1 diabetes 159, 164.

A drawback of C-peptide in clinical applications is its short circulating half-time (approximately 30 min). However, two approaches-PEGylation and conjugation with a thermosensitive biopolymer-have addressed this limitation. Wahren and colleagues 165 reported a PEGylated C-peptide, extending the half-life of C-peptide into 6-7 days, and applied this C-peptide to a clinical trial involving 250 patients with type 1 diabetes and peripheral neuropathy. Results indicate that subcutaneous administration of this PEGylated C-peptide for 52 weeks improves the vibration perception threshold but does not improve sural nerve conduction velocity, suggesting that C-peptide may not fully restore hyperglycemia-induced neuronal damage. Ha and colleagues 166 designed a controlled releasable human C-peptide, K9-C-peptide, by recombinantly conjugating human C-peptide with a lysine-containing elastin-like polypeptide. Subcutaneous injection of K9-C-peptide gradually releases human C-peptide from a hydrogel depot into circulation for 19 days, ameliorating aortic dysfunction in diabetic mice 167. K9-C-peptide simultaneously attenuates hyperglycemia-induced retinal, pulmonary, and glomerular dysfunctions in type 1 diabetic mice 28. Moreover, intravitreal injection of K9-C-peptide maintains physiological C-peptide levels in the intraocular space for at least 56 days, normalizing diabetic retinal neovascularization 74.

#### 4.2.2. Midazolam

Midazolam, a short-acting benzodiazepine medication widely employed for anesthesia, procedural sedation, and anxiolysis 168, demonstrates notable benefits against diabetic complications, including DR 77, DP 89, and DN 93. This is achieved by inhibiting VEGF-induced elevation of intracellular Ca^2+^, subsequent ROS generation, and TGase2 activation in a GAVA_A_ receptor-dependent manner **(Table 4)**.

In the retina of type 1 diabetic mice, intravitreal injection of midazolam mitigates hyperglycemia-induced vascular leakage by inhibiting ROS-mediated TGase2 activation. These inhibitory effects are reversed with flumazenil, a GAVA_A_ receptor antagonist 77. Subcutaneous injection of midazolam attenuates hyperglycemia-induced microvascular leakage and cancer metastasis by inhibiting TGase2-mediated VE-cadherin disassembly in the lungs of type 1 diabetic mice 89. Moreover, subcutaneous injection of midazolam in type 1 diabetic mice improves hyperglycemia-induced glomerular dysfunction, such as pathological alterations in glomerular ultrastructure and renal fibrosis.

While midazolam holds promise as a potential therapy for diabetic complications, the optimization of dosage and delivery routes is imperative to minimize potential side effects, including allergenic effects and dose-related respiratory depression. Careful consideration of these factors is essential for harnessing the full therapeutic potential of midazolam in mitigating the diverse complications associated with diabetes.

#### 4.2.3. Other ligands

A couple of ligands, including pigment epithelium-derived factor (PEDF), norrin, and somatostatin, have potential for therapeutic intervention of diabetic vascular complications, although their action mechanisms involving TGase2 remains unclear. PEDF is a member of the serine protease inhibitor superfamily with multiple biological functions and is known as an anti-angiogenic and neurotropic factor 169. PEDF binds to its multiple receptors, including laminin receptor, F1-ATP synthase, and low-density lipoprotein receptor-related protein 6, and exerts protective effects against diabetic microvascular complications by inhibiting oxidative stress and inflammation in the retina and kidney. 74, 170.

Norrin, a secreted 131-amino acid protein, utilizes the Wnt signaling pathway by binding to the frizzled class receptor 4 and the low-density lipoprotein receptor-related protein 5/6 co-receptor 171. Norrin attenuated hyperglycemia-induced vascular leakage by restoring disrupted blood-retinal barrier properties in diabetic retinas 171, 172. Somatostatin, which is a neuroprotective peptide, also ameliorated hyperglycemia-induced inflammation and neurodegeneration in the retina of diabetic rodent models 173, 174. However, further investigations to elucidate the underlying mechanisms of their beneficial effects can warrant clinical application for diabetic complications.

## 5. Conclusion and future perspectives

TGase2 stands as a multifunctional enzyme with transamidase, serine/threonine kinase, disulfide isomerase, and GTPase activities. Its non-enzymatic functions involve interacting with extracellular proteins, enhancing its role across various diseases, encompassing neurodegenerative disorders, pulmonary and kidney fibrosis, tumor initiation and progression, inflammatory conditions, and diabetic complications. This comprehensive review underscores TGase2's pivotal involvement in the pathogenesis of diabetic complications, including DR, DP, DN, CVD, and HGM. The proposed therapeutic strategies involve TGase2 inhibition, offering a promising avenue for managing diabetic complications.

Two primary approaches exist for TGase2 inhibition: direct and indirect. The direct inhibition strategy encompasses three groups-competitive, reversible, and irreversible inhibitors-each strategically impeding substrate binding to the TGase2 active site. Additionally, indirect TGase2 inhibition involves ligands binding to specific receptors, orchestrating TGase2 modulation through intricate intracellular signaling pathways. While both approaches hold considerable promise for treating diabetic complications, their clinical viability hinges on meticulous assessments of safety, efficacy, and possible adverse effects.

Direct TGase2 inhibitors exhibit distinct potential, with cystamine and its reduced form, cysteamine, emerging as particularly promising candidates. These amine inhibitors showcase compelling protective effects in preclinical studies, specifically ameliorating diabetic vascular dysfunctions in various organs, including the aorta, eye, lung, and kidney. Notably, these inhibitors have undergone clinical trials, demonstrating safety in conditions such as cystinosis and neurodegenerative disorders 120, 175.

Indirect TGase2 inhibition via ligands presents an attractive, comparatively safe avenue for treating diabetic complications. The proinsulin C-peptide stands out as an encouraging candidate, demonstrating beneficial effects against diabetic microvascular and macrovascular dysfunctions in both type 1 diabetic patients and animal models. The inherent endogeneity of human C-peptide secreted from pancreatic β-cells suggests minimal side effects. Further potential enhancements through PEGylation or conjugation with a thermosensitive polymer open avenues for extending its therapeutic half-life. Although human C-peptide may not fully address diabetic peripheral neuropathy, rigorous clinical trials are imperative to evaluate its efficacy in mitigating diabetic vascular complications. Similarly, midazolam, a short-acting benzodiazepine, shows promise, especially in treating DR through localized delivery. However, optimizing dosage and delivery routes and ensuring safety warrant exploration through clinical studies.

Clinical utilization of TGase2 inhibitors for diabetic complications necessitates an intricate understanding of TGase2's four enzymatic activities in the pathogenesis of hyperglycemia-induced vascular and neuronal dysfunctions. TGase2 transamidase activity orchestrates complex interactions with key proteins, including RhoA, NFκB, GAPDH, and E-cadherin, resulting in actin cytoskeleton rearrangement, inflammation, and disrupted energy metabolism, which are crucial factors in hyperglycemia-induced vascular dysfunctions. The TGase2 kinase activity, with its extensive substrate phosphorylation repertoire including NFκB, pRB, E-cadherin, and p53 oncoprotein, demands further study to elucidate its specific functions in diabetic complications. A nuanced understanding of the reciprocal regulation of vascular dysfunction-associated proteins by TGase2 transamidase and kinase activities introduces a compelling layer to the investigation. Furthermore, untangling the functions of TGase2 disulfide isomerase and GTPase activities introduces complexity, urging further investigations to unravel their roles in the pathogenesis of diabetic complications. The challenge and excitement lie in deciphering these intricate facets, expanding our understanding, and paving the way for potential therapeutic interventions that could transform the landscape of diabetic care.

## Figures and Tables

**Table 1:** Regulation of TGase2 transamidase and kinase activities.

Regulators	Transamidase	Kinase
Calcium	Activation 11	Inhibition 52, 54
GDP/GTP	Inhibition 176, 177	No effect 54
ATP	Inhibition 14, 52	Activation 14, 32, 52
Cystamine	Inhibition 14	Activation 14
Amine compounds^a^	Inhibition 17, 37, 38	N.D.
Thiol compounds^b^	Activation 14	Inhibition 14, 32
Nitric oxide	Inhibition 39	N.D.
Mg^2+^	No effect 14	Activation 14
Divalent metal cations^c^	N.D.	Inhibition 32
Phosphorylation	Inhibition 14	Activation 14
Acetylation	No effect 14, inhibition 42	Activation 14
C277S mutation	Complete inhibition 41, 178, 179	No effect 41
K444A mutation	Complete inhibition 41	Partial inhibition 41
K663A mutation	No effect 41	Partial inhibition 41

^a^putrescine, monodansylcadaverine (MDC), 5-(biotinamido)pentylamine (BAPA), spermine, spermidine, and histamine; ^b^glutathione, dithiothreitol, mercaptoethanol, and S-nitroso-N-acetylpenicillamine; ^c^Cu^2+^, Mn^2+^, Ni^2+^, and Zn^2+^; N.D.; not determined.

**Table 2:** Substrate proteins of TGase2 transamidase and kinase: functions and regulatory actions.

Substrate proteins	Transamidase activity	Kinase activity
RhoA	Activation: cell differentiation 43, stress fiber formation 48	N.D.
NF-κB	Activation by IκB polymerization: inflammation 44	Activation: PTEN downregulation and anti-apoptosis in cancer 56
GAPDH	Inhibition: interfering in energy metabolism 3, 45, 50; deamination: actin cytoskeleton remodeling 180	N.D.
pRB	Protection of pRB degradation: anti-apoptosis 33, 46, 48	Phosphorylation: disturbing E2F1 interactions and pro-apoptosis 52
E-cadherin	Transamidation 33	Phosphorylation: ECM breakdown and metastasis 30, 64
p53 oncoprotein	Inhibition: tumorigenicity 47	Phosphorylation: pro-apoptosis 58
IGFBP-3	N.D.	Phosphorylation: regulation of IGF functions 54
Histone proteins	N.D.	Phosphorylation: regulation of chromatin structure and function 53
G6PD	N.D.	Phosphorylation: oxidative stress 30
Cytochrome C	N.D.	Phosphorylation: pro-apoptosis 30, 61
Calmodulin	N.D.	Phosphorylation: Ca^2+^ signaling 30, 62
S100A7	Small effect 33	Phosphorylation: Ca^2+^ signaling 30
cMMP-3	N.D.	Phosphorylation: ECM breakdown and metastasis 30, 63

cMMP-3, catalytic domain of human matrix metalloproteinase-3; ECM, extracellular matrix; GAPDH, glyceraldehyde 3-phosphate dehydrogenase; G6PD, glucose 6-phosphate dehydrogenase; IGF, insulin-like growth factor; IGFBP-3, insulin-like growth factor-binding protein-3; NFκ-B, nuclear factor-κB; pRb, retinoblastoma protein; PTEN, phosphatase and tensin homolog; S100A7, S100 calcium-binding protein A7; N.D., not determined.

**Table 3:** TGase2 inhibitors for the treatment of diabetic complications.

Group	Inhibitor	Potential target diseases or functions
Monoamines	Cysteamine	Diabetic retinopathy 25, cystinosis 117, neurodegenerative diseases 119, 120
	Monodansylcadaverine	Diabetic retinopathy 24, 77, pulmonary disease 4, nephropathy 93
	Dopamine	Diabetic retinopathy 113, 124, 125
	5-(biotinamido)pentylamine	TGase pseudosubstrate 24, 27, 28, 33, 126, 127, 129
Polyamines	Cystamine	Diabetic retinopathy 24, 71, pulmonopathy 4, 78, nephropathy 89, 93, aortic dysfunction 65
	Putrescine	β-cell function 135, TGase substrate 133
	Spermidine	β-cell function 135, TGase substrate 133
	Spermine	β-cell function 135, TGase substrate 133
Irreversible inhibitors	Dihydroisoxazole derivatives	Anti-inflammation 141, antioxidant 144, glioblastoma 142, 143
	Michael acceptors	β-cell function 181, Celiac disease 137, neurodegenerative diseases 116
	Gluten peptide analogs	Celiac disease 150
	Acetylsalicylic acid	Acetylation of lysine residues 14, 42
Reversible inhibitors	LDN27219	Vascular dysfunction 154
	TGase2 siRNA	Diabetic retinopathy 24, pulmonary disease 4, nephropathy 93

LDN27219, thieno[2,3-d]pyrimidin-4-one acylhydrazides.

**Table 4:** Therapeutic agents for diabetic complications.

Inhibitor	Diabetic complication	Study models	Delivery system
Human C-peptide	Retinopathy	HRECs 24, type 1 diabetic mice 24, 71	Intravitreal injection 24, Osmotic pumps 71
	Pulmonopathy	HPMECs 4, type 1 diabetic mice 4, 78	Osmotic pumps 4, 78
	Nephropathy	Type 1 diabetic mice 27, type 1 diabetic patients 182	Osmotic pumps 27, subcutaneous injection 182
	Cardiovascular disease	HAECs 65, type 1 diabetic mice 27, 65	Osmotic pumps 27, 65,
	Peripheral neuropathy	Type 1 diabetic patients 182, 183	Subcutaneous injection 182, 183
PEG-C-peptide	Peripheral neuropathy	Type 1 diabetic mice 184, type 1 diabetic patients 165, 185	Subcutaneous injection 165, 184, 185
K9-C-peptide	Retinopathy	HRECs 28, type 1 diabetic mice 28, 74	Intravitreal injection 74, subcutaneous injection 28
	Pulmonopathy	Type 1 diabetic mice 28	Subcutaneous injection 28
	Nephropathy	Type 1 diabetic mice 28	Subcutaneous injection 28
	Cardiovascular disease	Type 1 diabetic mice 167	Subcutaneous injection 167
Midazolam	Retinopathy	HRECs 77, type 1 diabetic mice 77	Intravitreal injection 77
	Pulmonopathy	HPMECs 89, type 1 diabetic mice 89	Subcutaneous injection 89
	Nephropathy	HGMECs 93, type 1 diabetic mice 93	Subcutaneous injection 93

HAECs, human aortic endothelial cells; HGMECs, human glomerular microvascular endothelial cells; HPMECs, human pulmonary microvascular endothelial cells; HRECs, human retinal endothelial cells; K9-C-peptide, human C-peptide conjugated with nine repeats of lysine-containing elastin-like polypeptide; PEG-C-peptide, PEGylated human C-peptide.
